# The brazilian women in urology: current profile based on a practitioner's query

**DOI:** 10.1590/S1677-5538.IBJU.2020.1052

**Published:** 2021-03-05

**Affiliations:** 1 Centro Universitário Saúde ABC Departamento de Urologia Santo AndréSP Brasil Departamento de Urologia do Centro Universitário Saúde ABC, Santo André, SP, Brasil; 2 Hospital Moinhos de Vento Porto AlegreRS Brasil Serviço de Urologia do Hospital Moinhos de Vento, Porto Alegre, RS, Brasil

## COMMENT

Historically, urology and orthopedics are male medical specialties. The Brazilian Urologic Society (SBU) database shows 4.621 male and 125 female board-certified urologists (or 37-1 male-female ratio). This predominant male scenario has changed in the last decade, with a significant increase in female urologists. However, specific barriers hinder further expansion of female urologists within the specialty (1). One of women's challenges in a predominantly male environment is the lack of guidance from female leadership models and the opportunity for relevant academic positions.

This research project aims to evaluate the profile of female urologists in Brazil through the on-line submission of an SBU-approved questionnaire to all board-certified urologists and urology residents. The questionnaire included the following items: date of birth, federative state of operation, professional training (whether residents, active, or retired professionals), seniority in the field - in pre-defined time frames (residents, 1-2 years, 2-5 years, 5-10 years, and more than 10 years) and their area of activity, whether clinical or surgical urology (or both) and subspecialty (urology residents were oriented to skip this item).

The results were tabulated in a Microsoft Excel spreadsheet. Continuous variables were presented as mean and standard deviation (SD). Frequency distributions (absolute and relative) and proportions were presented (in a table or a graph, including pie charts) for categorical variables. The SPSS v.23.0 (IBM Corp., Armonk, NY, USA) was employed for the analyses.

According to the Resolution of the Brazilian National Health Council nº510/2016 that addresses Human and Social Sciences Research, “VII research that aims to deepen the theoretical hypotheses that emerge spontaneously and contingently in professional practice, as long as they do not reveal data that identify the subject” will not be provided or evaluated by the CEP/CONEP system. It is important to note that all the required measures were adopted to enforce the basic solution's ethical recommendations. Producing guidelines for the category is a condition of the medical societies planned in their establishment rules. Therefore, it is necessary to collect information about professional practice. Thus, it is legitimate for the Brazilian Society of Urology to ask the urologists about their practice.

A total of 129 women, including urologists and residents, completed the questionnaire from August 16 to 24, 2020. The average age was 37.2 years (27 to 68), 43 (33.3%) women were from São Paulo, 19 (14.7%) from Rio de Janeiro, and 13 (12.4%) from Rio Grande do Sul. Notably, 78 (60.1%) of all women were from three federation states, with no representatives from Paraíba, Mato Grosso do Sul, Acre, Alagoas, Roraima, Rondônia, Amapá, Sergipe, and Tocantins.

**Figure 1:**
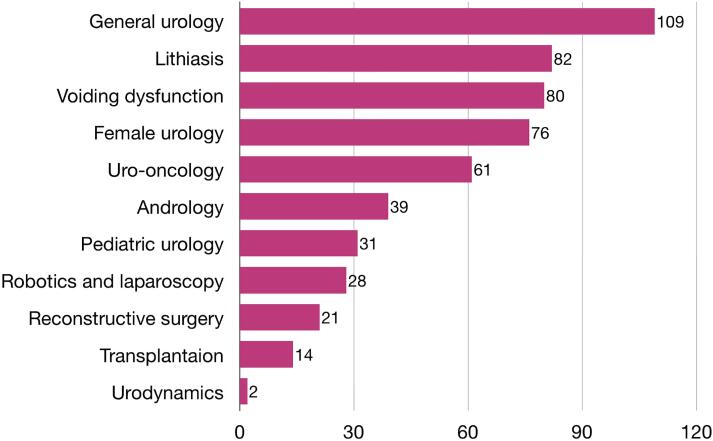
Subarea of urological expertise reported by the participants.

Ninety (69.75%) of the 129 women included in this analysis were board-certified urologists actively working in the field, 38 (29.45%) were urology residents, and 1 (0.8%) were retired urologists. Regarding seniority since the urology board certification, 24 (18.6%) had less than 2 years, 21 (16.3%) 2 to 5 years, 22 (17.05%) 5 to 10 years, and 24 (18.6%) had 10 or more years’ experience (38 women (29.4%) were still in residency). When these 129 women were asked about their current situation, 92.2% defined themselves as clinical and surgical urologists, while 7.8% as clinical urologists only. The subspeciality distribution was as follows: 109 (84%) general urology, 82 (63%) lithiasis, 80 (62%) voiding dysfunction, 76 (58%) female urology, 61 (47%) uro-oncology, 39 (30%) andrology, 31 (24%) pediatric urology, 28 (21%) robotics and laparoscopy, 21 (16%) reconstructive surgery, 14 (10.8%) transplantation, and 2 (1%) urodynamics. The subspecialty distribution is depicted in Figure-1.

Based on the questionnaire data, the largest concentration of female urologists is in the state of São Paulo, followed by Rio de Janeiro and Rio Grande do Sul. The lack of female urologists in North and Northeast regions of Brazil is a critical issue to be addressed in the national context. Regarding urology subspecialties, most women are subspecialized in lithiasis, voiding dysfunction, and female urology. Given that most urologic patients are men, the subspecialty distribution may be related to taboos or a gender preference for urologists, as observed in a research conducted in the U.S., where women would prefer same-gender doctors to perform their treatment. Thus, the choice of subspecialties that involve women may also correspond to market demand (2, 3). Notably, the number of female urologists is on the rise in Brazil, just like in other countries like New Zealand, Australia, and the U.S. (4, 5). Given the increase of this professional group, societies must be aware of their needs and necessities to further strengthen them daily.
